# Mitochondrial Drugs for Alzheimer Disease

**DOI:** 10.3390/ph2030287

**Published:** 2009-12-23

**Authors:** David J. Bonda, Xinglong Wang, Katarzyna A. Gustaw-Rothenberg, George Perry, Mark A. Smith, Xiongwei Zhu

**Affiliations:** 1 Department of Pathology, Case Western Reserve University, Cleveland, OH 44106, USA; 2 Memory and Cognition Center, University Hospitals Case Medical Center, Beachwood, OH 44122, USA; 3 Department of Neurodegenerative Diseases, Institute of Agricultural Medicine, Lublin, Poland; 4 UTSA Neurosciences Institute and Department of Biology, College of Sciences, University of Texas at San Antonio, San Antonio, TX 78249, USA

**Keywords:** Alzheimer disease, antioxidant, coenzyme Q, Dimebon, fission, fusion, mitochondria, mitochondrial drugs, mitochondrial permeability transition pore, oxidative stress

## Abstract

Therapeutic strategies for Alzheimer disease (AD) have yet to offer a disease-modifying effect to stop the debilitating progression of neurodegeneration and cognitive decline. Rather, treatments thus far are limited to agents that slow disease progression without halting it, and although much work towards a cure is underway, a greater understanding of disease etiology is certainly necessary for any such achievement. Mitochondria, as the centers of cellular metabolic activity and the primary generators of reactive oxidative species in the cell, received particular attention especially given that mitochondrial defects are known to contribute to cellular damage. Furthermore, as oxidative stress has come to the forefront of AD as a causal theory, and as mitochondrial damage is known to precede much of the hallmark pathologies of AD, it seems increasingly apparent that this metabolic organelle is ultimately responsible for much, if not all of disease pathogenesis. In this review, we review the role of neuronal mitochondria in the pathogenesis of AD and critically assess treatment strategies that utilize this upstream access point as a method for disease prevention. We suspect that, with a revived focus on mitochondrial repair and protection, an effective and realistic therapeutic agent can be successfully developed.

## Introduction

Mitochondrial abnormalities have long been implicated in the aging process, but only recently has their influence been extended to neurodegenerative disease [1,2,3,4]. Alzheimer disease (AD), in particular, appears to involve the eventual and progressive dysfunction of neuronal mitochondria; such mitochondrial aberration in fact seems to elicit the hallmark pathologies of the disease, notably amyloid-β (Aβ) plaques and hyperphosphorylated microtubule-associated protein tau in the form of neurofibrillary tangles (NFTs), and ultimately seems responsible for the characteristic neurodegeneration found in AD [5]. Specifically, mitochondria are associated with neurodegeneration in AD for several reasons, including: (1) They are the primary generators of reactive oxidative species (ROS) within the cell [3]; (2) Damage to mitochondrial structural components and enzyme complexes are well documented in AD and vastly precede any other hallmark feature of the disease [6,7,8,9]; and (3) Mitochondrial dynamics have been demonstrated as severely altered in AD neurons when compared to controls [1,5,10,11]. Since AD is the leading cause of senile dementia in the United States, affecting 15% of people over the age of 65 and almost 50% of those over 85 [12], the need for an effective preventative measure against disease onset and progression is ever increasing [13], and we here present current perspectives on mitochondrial drugs for neurodegeneration in an attempt to further therapeutic scrutiny. 

## Mitochondrial ROS Generation and Oxidative Stress in Alzheimer Disease: An Opportunity for Intervention

Aerobic respiration, accounting for 95% of the animal cell’s energy supply, inevitably produces reactive oxidative species within the cell. Although there are mechanisms in place to sequester such oxidants before they wreak internal havoc, the citric acid (TCA) cycle and the process of oxidative phosphorylation (both occurring within mitochondria) undoubtedly generate a surplus of free radicals every day. In fact, some estimates suggest a daily production of 10^11^ ROS within a typical aerobic cell [14]. As the brain uses approximately 20% of the body’s oxygen supply despite only comprising 2-3% of the body’s mass [14], a typical adult neuron certainly generates more. While these ROS are manageable in a young, healthy cell, eventually, their accumulation leads to cellular detriments that futher the cell’s inability to defend itself. Consequently, oxidative stress becomes a significant role player in cellular dysfunction within the brain, and, in fact, it has been well documented in neurodegenerative diseases, particularly that of AD.

The “two-hit hypothesis” for AD describes a phenomenon whereby the oxidative stress elicited by dysfunctional respiratory processes (hit one) produces compensatory changes in the cell that enable it to function for decades under said stress [8,15,16]. Unfortunately, these compensatory changes, including the deposition of Aβ and phosphorylation of tau as an antioxidant [17,18], make the cell vulnerable to additional insults, such as mitotic aberrations (hit two), and it is the cell’s inability to defend itself against these second “hits” due to the compensatory “steady state” that ultimately ensures its demise. Notably, once oxidative damage within the cell initiates these changes (*i.e.,* after years of accumulation and slow oxidative damage), a vicious cycle ensues in which Aβ produces neuroinflammation and microglial activation which, in turn, elicits further oxidative stress via damage to vital mitochondrial components, such as pyruvate dehydrogenase complex (PDHC), ketoglutarate dehydrogenase complex (KGDH), and cytochrome *c* oxidase (COX) [1,19,20,21,22]. As any disturbance in the respiratory enzyme complexes further generates ROS, because the mitochondrial respiratory proteins orchestrate the oxidation/reduction reactions that enable the generation of ATP, any perturbance in their mechanisms undoubtedly leads to additional damage [23,24,25,26,27].

Additional insults to mitochondrial components, resulting from oxidative stress, have also been indentified in AD, including altered Ca^2+^ homeostasis (due to mitochondrial impairment) and mitochondrial DNA (mtDNA) mutations and deletions [9,28,29]. As the mitochondria are the initial producer of ROS, and thus of neuronal oxidative damage, and because mitochondria are so vulnerable to oxidative damages once oxidative stress becomes uncontrollable, recent studies have turned to antioxidant therapies in hopes of providing a preventative cure for AD [30,31,32]. Mitochondrial antioxidants are of particular appeal and have provided the greatest evidence of laboratory efficacy.Coenzyme Q_10_ (CoQ_10_) administration, for example, has been shown to elicit neuroprotective effects in AD by nullifying oxidative damage and attenuating mitochondrial dysfunction [3,33]. Functionally, CoQ_10_ is an electron carrier in the electron transport chain (ETC) of oxidative phosphorylation that is embedded in the inner mitochondrial membrane. It acts to carry the high energy electrons in the chain from complex I to complex II, and its redox cycling from the oxidized form (ubiquinone) to the reduced form (ubiquinol) is dependent on ETC functioning [34,35]. A recent report indicated that a dose of 6.5 μM CoQ_10_ to MC65 neuroblastoma cells provided complete protection from neurotoxicity due to oxidative stress, and suppressed H_2_O_2_ and O_2_^-^ production [36]. Although brain tissue and mitochondrial levels of CoQ_10_ were not increased by oral administration (confirmed by other reports [37,38,39], oxidative damage to brain proteins was attenuated [36]. Importantly, the inability of orally administered antioxidants to breach the blood brain barrier (BBB) presents a hurdle in the development of effective neurodegeneration treatments, and has led to the investigation of more soluble, shorter chain antioxidant CoQ_10_ derivatives, such as idebenone (6-(10-hydroxydecyl)-2,3-dimethoxy-5-methyl-1,4-benzoquinone) and decylubiquinone (dUb) [40,41]. Additionally, mitochondria-targeted antioxidants have been investigated that further increase specificity of ROS sequestration in neurodegeneration, the most successful of which has been MitoQ, a triphenylphosphonium-linked ubiquinone derivative [42].

**Figure 1:**
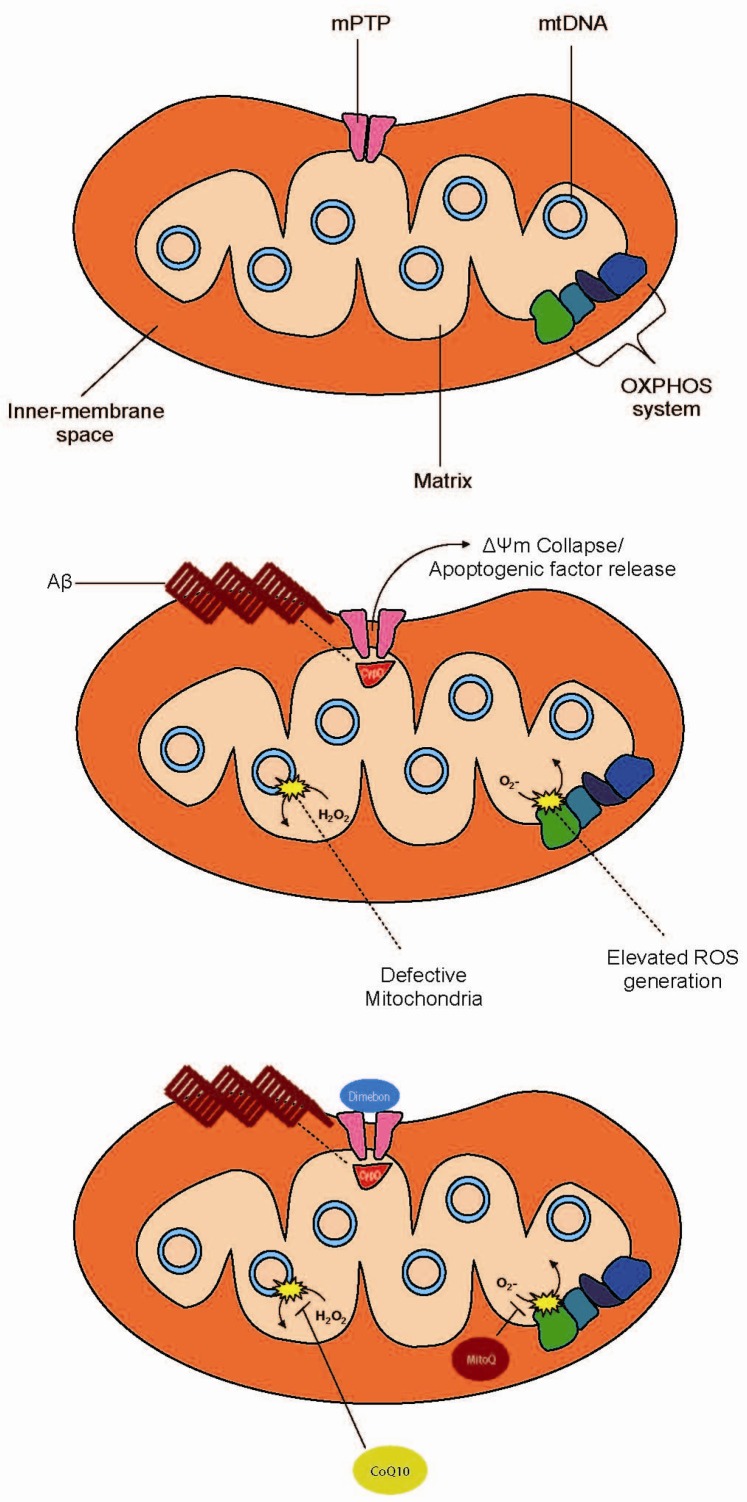
(A) A mitochondrion with intact components typical of healthy brain: functional oxidative phosphorylation (OXPHOS) system, non-mutated mtDNA, closed, impermeable mPTP. (B) In an aging, AD-prone brain, mitochondrial dysfunction mediates much of the characteristic neurodegeneration. Free radical accumulation produces damages to mtDNA and the OXPHOS system, eliciting further oxidative stress; As Aβ aggregates, it promotes cycliphilin D (cypD)-induced mitochondrial permeability transition pore (mPTP) opening (shown by the dotted line connecting them), collapsing mitochondrial membrane potential (Δψm) and releasing apoptogenic factors. (C) Mitochondrial intervention thus provides an adequate therapeutic access point to AD prevention and control: the antioxidants MitoQ and CoQ10 are under investigation as is the mPTP protector Dimebon.

MitoQ is known to concentrate within mitochondria (several hundred-fold) due to the large mitochondrial membrane potential [43]. Its selective accumulation in the metabolic organelle, and its continual recycling by mitochondrial enzymes (including those of the ETC), make MitoQ a much more potent antioxidant than those that are non-targeted [44]. Indeed, studies have confirmed the beneficial role of MitoQ in neurodegenerative models [40,45]. Specifically, in parental leukemic CEM cell cultures, the effects of MitoQ were demonstrated to be remarkably protective after depletion of glutathione (GSH), a regulator of mitochondrial permeability transition [40]. That is, MitoQ: (1) effectively blocked ROS generation; (2) protected mitochondrial protein redox status; (3) preserved the integrity of mitochondrial structures; and (4) blocked cell death after depletion of GSH [40]. Moreover, MitoQ was determined to be a more effective antioxidant for mitochondria, when compared to CoQ_10_, and was demonstrated to be effective in the absence of a functioning ETC [40]. While these data correspond to cell cultures and non-neuronal tissues, they nonetheless indicate the potential benefits of MitoQ in the treatment of oxidative stress-related disease. In fact, MitoQ is currently under development in phase II clinical trials for Parkinson’s disease and liver damage associated with HCV infection [45], and the results will hopefully shed light on the applicability of the drug to other diseases, such as AD (Figure 1).

## Mitochondrial Permeability Transition Pore: A Gateway to Stress Relief

In addition to oxidative stress, the deterioration of neuronal mitochondria occurs via alterations in the mitochondrial permeability transition pore (mPTP), the transmembrane protein complex responsible for regulating the permeability of the inner mitochondrial membrane. While this aspect of neurodegeneration is downstream from the aforementioned mitochondrial stresses (mPTP breach occurs due to Aβ-induced stresses [46]), it nonetheless exists as an integral part of the cycle toward neuronal decay. Specifically, deficiencies in the mPTP elicit collapses in mitochondrial membrane potential, which renders the mitochondria entirely dysfunctional [46], and amplify apoptotic mechanisms by releasing protein with apoptogenic potential from the inner membrane space [47,48,49].

Structurally, the mPTP is composed of a voltage-dependent anion channel in the outer membrane, the adenine nucleotide translocase protein in the inner membrane, and the cyclophilin D (cypD) protein in the matrix [48,50,51,52,53]. The latter protein, cypD, is a peptidylprolyl isomerase F that resides in the mitochondrial matrix and associates with the inner mitochondrial membrane during mPTP opening [46]. Interestingly, it seems as though cypD is necessary for pore opening and that oxidative (as well as other) cell stresses trigger its translocation to the inner membrane, thus opening the pore [54,55,56,57,58,59]. Studies correspondingly show that a genetic deficiency in cypD protects neurons from Ca^2+^- and oxidative stress-induced cell death [58]. Moreover, Aβ has been demonstrated to bind to and associate with cypD, thus providing a mechanistic link to Aβ, mitochondrial dysfunction, and neurodegeneration [60,61,62], and the absence of cypD has similarly been shown to protect neurons from Aβ-induced death [46]. cypD deficiency also improved behavioral and synaptic function in rats [46]. While these reports only elucidate the role of cypD in AD-type neurodegeneration and do not describe any specific drug, they do indicate an excellent opportunity for therapeutic intervention.

Notably, studies of the antihistamine drug Dimebon (2,3,4,5-tetrahydro-2,8-dimethyl-5[2-(6-methyl-3-pyridinyl)ethyl]-1H-pyrido[4,3-b]indole) indicate its ability to block mPTP opening and protect against oxidative/Aβ-induced cellular dysfunction and death [63,64,65,66]. Dimebon has also been demonstrated to act as an antagonist to NMDA receptors and as a blocker of Ca^2+^ channels, in both cases providing protection from excitotoxicity (another common feature of AD neurodegeneration) [63,64,67]. Specifically, open label clinical trials in the Moscow Center for Gerontology on 14 patients with mild to moderate AD demonstrated significant progressive improvement in the cognitive functions of all patients [63]. Additionally, a reduction of neuropsychiatric symptoms was demonstrated [63]. Another study reported on a randomized, double blind, placebo-controlled experiment on patients with mild to moderate AD and revealed that Dimebon significantly improved five measures of patient brain health over the course of the entire study, including cognition (MMSE and ADAS-cog), function (ADCS-ADL), and behavior (NPI) [67]. Drug safety was confirmed in both studies as the drug produced little to no adverse side effects, and patients tolerated the drug especially well [63,67]. Again, although there is much more investigation necessary on Dimebon before we completely understand the mechanism of action, these results provide an attractive and excellent treatment possibility that should certainly be explored (Figure 1).

## Mitochondrial Dynamics: Fission, Fusion, and Neurodegeneration

Mitochondria are dynamic organelles that constantly divide and fuse within the cell as its varying energy demands require [68]. Each process (*i.e.,* that of fission and fusion) is controlled by particular membrane-embedded protein complexes that coordinate the appropriate dynamic steps; a disruption in any such complex elicits abnormal mitochondrial dynamics and devastates the cell. In AD, an alteration of mitochondrial dynamics is indeed evident [1,5,10], and its role in disease pathogenesis is becoming increasingly apparent. As such, a therapeutic intervention that corrects the abnormal mitochondrial dynamics of AD is an exciting possibility.

Within a healthy neuron, a delicate balance of fission and fusion is necessary for cellular integrity [69,70,71,72,73,74]. In particular, a genetic inactivation of fusion yields abnormal mitochondrial fragmentation [75] and that of fission results in mitochondrial elongation [76]. Importantly, these processes are the cell’s greatest defense against the catastrophic feedback cycle that is described above as typical of AD. That is, they prevent mitochondrial abnormalities from accumulating in the cell to yield vast oxidative damage and neuronal death. Fusion, for example, allows the exchange of lipid membrane and inter-mitochondrial components (*i.e.,* mtDNA and fission/fusion proteins) thus lowering the percentage of defective mitochondria within the cell [77,78,79]. Fission, on the other hand, coupled with fusion and autophagy, enables the sequestration and elimination of irreversibly damaged mitochondria and mitochondrial content [77,78,79]. Consequently, any damage incurred onto mitochondrial enzyme complexes or DNA (resulting from inevitably generated ROS within the cell) is dampened by the mitochondrial dynamic system.

Notably, however, over the span of years, and even decades, sporadic mutations in mtDNA, due to the aforementioned intracellular ROS, accumulate to eventually reach a threshold whereby the system of dynamics loses control. In this age-induced mitochondrial cascade, mtDNA mutations elicit dynamics alterations that then propagate the presence of defective mitochondria and further the production of ROS, oxidative stress, defective mitochondria, and so on. AD and its associated pathologies are thus the clinical manifestation of neuronal stresses that originate in the mitochondria. While these occurrences unfortunately seem to be an inevitable part of aging, our knowledge of the key components of the cycle (*i.e.,* mitochondria, mitochondrial dynamics proteins, mtDNA, *etc.*) allows another preventative access point for therapeutics that can hopefully dampen or eliminate the devastating process. While to our knowledge there has been no such development of treatment strategies that target mitochondrial dynamics proteins thus far, it is certainly an area that merits much attention for future research.

## Current Treatment Perspectives

Importantly, while the treatment strategies discussed above present appealing cases for neuroprotective agents against AD, they are far from becoming of widespread use. In fact, several limitations hinder their progress towards disease prevention. As to antioxidant strategies, the simple elimination of neuronal ROS *in vivo* is perhaps too little, too late. That is, the specific antioxidant pathways used, the use of ROS as signaling molecules, and the varying proteins involved in mitochondrial processes instill limitations to a general sequestration of all ROS by therapeutic agents as it may result in adverse side effects. While specifically targeted mitochondrial antioxidants, such as MitoQ, do represent progress in this aspect, their successful penetration into brain mitochondria has yet to be confirmed and presents additional challenges to its efficacy.

Additionally, the years of progressive detriment that characterizes ROS damage to neurons in AD, and that elicits AD pathologies such as Aβ plaques and NFTs, are most likely too severe to be fully attenuated, let alone reversed, by even the most specific antioxidant. By the time of disease diagnosis, for example (that is, after the clinical appearance of cognitive decline and AD psychological manifestations), drastic damage to the brain has already occurred well beyond anything subsequent antioxidant therapy could heal. Consequently, any mitochondrial antioxidant must be administered well before disease onset. Only then could ROS sequestration effectively prevent, or at least slow, the progression and onset of AD. Similarly, mPTP treatments present strategies that target deficiencies in mitochondria that occur years into the disease. Even more, although they are seemingly effective, they do not target the underlying cause of the problem (*i.e.,* mitochondrial ROS and dynamics alterations) but rather attenuate Aβ-induced mitochondrial stress. Ultimately, while much more investigation is necessary, it seems as though early treatment, combined with a targeting of underlying disease causes, is the most effective way to prevent AD. 
